# Descriptive figures for differences in parenting and infant night-time distress in the first three months of age

**DOI:** 10.1017/S1463423616000293

**Published:** 2016-09-09

**Authors:** Ian St James-Roberts, Marion Roberts, Kimberly Hovish, Charlie Owen

**Affiliations:** Thomas Coram Research Unit, UCL Institute of Education, University College London, 27/28 Woburn Square, London WC1H 0AA, UK

**Keywords:** infant crying, infant sleeping, parenting

## Abstract

**Aim:**

To provide descriptive figures for infant distress and associated parenting at night in normal London home environments during the first three months of age.

**Background:**

Most western infants develop long night-time sleep periods by four months of age. However, 30% of infants in many countries sleep for short periods and cry out on waking in the night: the most common type of infant sleep behaviour problem. Preventive interventions may help families and improve services. There is evidence that ‘limit-setting’ parenting, which is common in western cultures, supports the development of settled infant night-time behaviour. However, a recent review has challenged this and argued that this form of parenting risks distressing infants. This study describes limit-setting parenting as practiced in London, compares it with ‘infant-cued’ parenting and measures the associated infant distress.

**Methods:**

Longitudinal infrared video, diary and questionnaire observations comparing a General-Community (*n*=101) group and subgroups with a Bed-Sharing (*n*=19) group on measures of infant and parenting behaviours at night.

**Findings:**

General-Community parents took longer to detect and respond to infant waking and signalling, and to begin feeding, compared with the highly infant-cued care provided by Bed-Sharing parents. The average latency in General-Community parents’ responding to infant night-time waking was 3.5 min, during which infants fuss/cried for around 1 min. Compared with Bed-Sharing parenting, General-Community parenting was associated with increased infant distress of around 30 min/night at two weeks, reducing to 12 min/night by three months of age. However, differences in infant distress between General-Community subgroups adopting limit-setting versus infant-cued parenting were not large or statistically significant at any age. The figures provide descriptive evidence about limit-setting parenting which may counter some doubts about this form of parenting and help parents and professionals to make choices.

Most western infants develop long sleep periods in the night-time by four months of age (Moore and Ucko, 1957; Anders *et al*., 1992; Henderson *et al*., 2010). For example, around 60–70% of four-month olds meet the criterion of sleeping continuously for periods of 5 h or more most nights per week (Moore and Ucko, 1957; St James-Roberts *et al*., 2001; Henderson *et al*., 2010). However, around a third of infants in many countries sleep for short periods and ‘signal’ (cry out) on waking in the night, making this the most common type of infant sleep behaviour problem (Sadeh and Sivan, 2009; Mindell *et al*., 2010). Most such infants are healthy (Mindell, 2008; Sadeh and Sivan, 2009), but their night-time behaviour can exhaust or depress parents and generate substantial health service costs (Morris *et al*., 2001; Dennis and Ross, 2005; Stremler *et al*., 2013). Preventive interventions have the potential to help families and improve service cost-effectiveness.

Four randomised controlled trials (RCTs) found that ‘limit-setting’ parenting increased the number of infants with long night-time sleep periods at three to four months of age (Wolfson *et al*., 1992; Pinilla and Birch, 1993; St James-Roberts *et al*., 2001; Symon *et al*., 2005). This form of parenting is common in western societies, employing routines and delayed responding to encourage infants to develop autonomous settling (Jenni and O’Connor, 2005; St James-Roberts *et al*., 2006). In contrast, ‘infant-cued’ parenting, including high proximity, rapid responses and, in some cases, bed-sharing, is associated with persistent infant waking and signalling at night (St James-Roberts *et al*., 2006; Sadeh *et al*., 2010; Hysing *et al*., 2014). Importantly, limit-setting parenting reduced breast-fed infants’ night-time signalling by four months without affecting weight gain (Pinilla and Birch 1993; St James-Roberts *et al*., 2001), indicating that it is compatible with breast-feeding.

Unfortunately, two recent attempts to apply these findings to community health services did not increase the numbers of infants with long sleep periods at night (Stremler *et al*., 2013; Hiscock *et al*., 2014). A possible explanation is that limit-setting parenting is common in the communities involved, so that intervention and control groups were similar (Stremler *et al*., 2013). However, a recent review has concluded that limit-setting parenting does not support sleep-waking development and risks increasing infant distress (Douglas and Hill, 2013).

This study’s aim was to address these concerns by providing descriptive evidence about night-time parenting and infant distress in London: a western society known to favour limit-setting care (St James-Roberts *et al*., 2006). For comparative purposes, a group of parents who planned to bed-share with their babies, and were expected to adopt infant-cued parenting, was included.

Medical Research Council guidelines for evaluating complex interventions stress the need for observational studies to support RCTs (Medical Research Council (MRC), 2007). Although they cannot prove causation, observational studies are superior at documenting what typically happens in participants’ normal environments. As well as parental measures, we used infrared video-recording methods developed by Anders and colleagues and proven valid for observation of night-time behaviours (Anders and Keener, 1985; Goodlin-Jones *et al*., 2001; Sitnick *et al*., 2008).

## Methods

### Participants

Participants were breast-feeding mothers and singleton infants living within the M25 motorway around London, UK. The ‘General-Community’ group (*n*=101) was recruited in postnatal wards of a community maternity hospital. We excluded multiple births, infants with birth weight <2500 g, admitted to special care or who medical staff considered unwell, and mothers with limited English. Otherwise, mothers were approached consecutively, introduced to the study, and asked to allow a telephone call to explain the research fully after returning home. Mothers who agreed gave written informed consent and completed the newborn Infant Sleep & Feeding Arrangements Questionnaire (ISFAQ) described below when infants were <48 h old.

For comparison, we recruited a group of mothers who planned to adopt infant-cued parenting. Prior studies showed that mothers who intend to bed-share with their babies are likely to adopt highly infant-cued parenting behaviours in general (St James-Roberts *et al*., 2006; Hysing *et al*., 2014). Mothers included in this study’s planned bed-sharing (‘Bed-Sharing’) group met the recruitment criteria of the General-Community group but intended to bed-share with their babies most of the night (defined as ⩾90% of the night, ⩾5 nights/week). Only one mother approached in the maternity hospital met these criteria; most (18 of 19) were recruited during pregnancy via parenting networks. They gave written informed consent and completed the ISFAQ before, or within 48 h of, their baby’s birth. This group’s size was small despite 18 months of recruitment, possibly because this coincided with medical guidance that bed-sharing is unsafe. Table 1 lists recruitment, participant and missing data.

**Table 1:** Descriptive figures for recruitment, attrition, participants’ characteristics and missing data

	General-Community group	Bed-Sharing group	Overall
Number approached (gave consent)	358 (213)	NA^a^	NA^a^
Number of participants^b^			
Newborn ISFAQ	101	19	120
Two-week ISFAQ and diary	101	19	120
Five-week ISFAQ/diary/video	101/100/99	19/18/18	120/118/117
Three-month ISFAQ, diary and video	101	19	120
Six-month ISFAQ and BISQ	93	18	111
Boys [*n* (%)]	57 (56.4)	8 (42.1)	65 (54.2)
Firstborns [*n* (%)]	72 (71.3)	6 (31.6)*	78 (65.0)
Infant ages [mean (SD)]			
Days at two-week assessments	11.5 (1.4)	12.0 (2.3)	11.6 (1.6)
Days at five-week assessments	37.9 (4.9)	40.6 (4.1)	38.3 (5.0)
Days at three-month assessments	89.3 (6.8)	92.6 (8.4)	89.8 (8.4)
Weeks at six-month assessments	27.8 (1.3)	28.7 (2.3)	28.1 (1.8)
Mother’s age in years [mean (SD)]	32.9 (4.4)	34.1 (4.6)	33.1 (4.5)
Father’s age in years [mean (SD)]	34.7 (5.0)	36.0 (3.9)	34.9 (4.9)
% mothers/fathers white ethnicity	85.1/82.0	94.7/94.4	86.7/83.9
% mothers/fathers with university education	75.2/77.0	89.5/88.2	77.5/78.6
% mothers employed full-time before maternity leave	62.4	42.1	59.2
% fathers employed full-time	89.0	88.9	89.0
% parents married/co-habiting/single parent	76.2/22.8/1	68.4/26.3/5.3	75.0/23.3/1.7
Number of rooms in home (excluding bathroom) [mean (SD)]	3.6 (1.5)	4.1 (1.9)	3.7 (1.6)

BISQ=Brief Infant Sleep Questionnaire; ISFAQ=Infant Sleep & Feeding Arrangements Questionnaire.

a Numbers not known: most bed-sharing parents contacted via parenting networks or websites.

b Missing data: at five weeks, no usable video data were obtained for one bed-sharing and two General-Community cases; no diary data were provided by one General-Community and one bed-sharing case. Diaries were for 72 h, except two diaries (48 h) were provided by four parents at 10 days, ten at five weeks and six at 12 weeks; two parents completed one 24 h diary at five weeks. Eight General-Community and one bed-sharing family did not return six-month data.

*Fisher’s exact test (two-sided) *P*<0.001.

### Newborn assessments

The newborn ISFAQ was constructed following earlier studies (Morrell, 1999; St James-Roberts *et al*., 2001; Tikotzky and Sadeh, 2009) to provide a brief screen for the mothers’ intended parenting strategies at home. Mothers answered seven questions by ticking boxes or inserting figures. Table 2 gives the wording of the ISFAQ items, including those used to assess the key limit-setting behaviours of response delay and feeding interval (items 6 and 7). Other items measured whether parents intended to use evening routines, such as bathing, to settle their baby (item 4) and whether they planned to settle their baby to sleep at a regular time or when tired (‘settling method’, item 5).

**Table 2:** Group descriptive figures at each age for the Infant Sleep & Feeding Arrangements Questionnaire measures

Groups	General-Community group					Bed-Sharing group				
Age	Newborn	Two weeks	Five weeks	Three months	Six months	Newborn	Two weeks	Five weeks	Three months	Six months
ISFAQ measures^a^										
1. How are you planning to feed (currently feeding) your baby?										
Breast only (%)	73.3	66.3	53.5	50.5	25.8	84.2	94.7	89.5	89.5	83.3
Breast+expressed breast (%)	24.8	12.9	17.8	13.9	11.8	15.8	5.3	10.5	10.5	11.1
Breast+formula (%)	2.0	18.8	22.8	24.8	34.4	0	0	0	0	0
Formula only	0	2.0	5.9	10.9	28.0	0	0	0	0	5.6
2. Where will (does) baby usually sleep at night?										
In parents’ bed (%)	0	6.9	6.9	3.0	5.4	100	100	94.7	84.2	66.7
Mattress/futon next to parents’ bed (%)	0	0	0	0	1.1	0	0	0	0	0
Cot next to parents’ bed (%)	71.3	69.3	53.5	41.6	19.4	0	0	5.3	5.3	11.1
Cot elsewhere in parents’ bedroom (%)	21.8	21.8	27.7	32.7	18.3	0	0	0	0	5.6
Cot in sibling’s room (%)	0	0	0	0	0	0	0	0	5.3	0
Cot in separate room (%)	6.9	2	11.9	22.8	55.9	0	0	0	5.3	16.7
3. Will (Does) baby ever sleep in bed with you?										
No (%)	53.5	55.4	49.5	60.4	63.4	0	0	5.3	5.3	11.1
Yes (%)	46.5	44.6	50.5	39.6	36.6	100	100	94.7	94.7	88.9
4. Are you planning to use (currently using) routines each evening – such as bathing at a regular time – to help baby to settle at night?										
No (%)	14.9	66.3	41.6	16.8	6.5	26.3	78.9	84.2	57.9	38.9
Yes (%)	53.5	33.7	58.4	83.2	93.5	47.4	21.1	15.8	42.1	61.1
Not thought about it (%)	31.7	0	0	0	0	26.3	0	0	0	0
5. Do you think it is best to put your baby down to sleep at the same time each night, or when he/she is tired and starts to fall asleep?										
Always settle when tired (%)	0	34.7	20.8	11.9	7.5	36.8	42.1	52.6	52.6	27.8
Almost always when tired (%)	5.9	12.9	9.9	12.9	7.5	31.6	21.1	15.8	10.5	5.6
Usually when tired (%)	8.9	5.9	9.9	5.9	4.3	0	15.8	15.8	10.5	11.1
Sometimes tired/sometimes at a regular time (%)	31.7	10.9	10.9	11.9	11.8	15.8	10.5	10.5	5.3	27.8
Usually same time (%)	33.7	16.8	28.7	27.7	21.5	10.5	5.3	5.3	15.8	22.2
Almost always same time (%)	15.8	12.9	16.8	26.7	37.6	5.3	5.3	0	5.3	5.6
Always same time (%)	4.0	5.9	3.0	3.0	9.7	0	0	0	0	0
6. If baby wakes in the night, how many minutes are you likely to leave him/her to cry before you pick him/her up?										
Minutes [mean (SD)]	2.15 (2.97)	1.70 (2.06)	2.54 (3.33)	3.00 (4.35)	7.47 (15.15)	0.32 (1.16)	0.58 (2.29)	0.05 (0.23)	0.79 (1.68)	0.64 (1.62)
Parents reported 0 min: will never leave him/her to cry (%)	34	40.6	28	33.7	26.7	89.5	89.5	94.7	78.9	83.3
7. If baby wakes in the night, how many minutes will (does) it take before you feed him/her?										
Minutes [mean (SD)]	2.23 (3.02)	3.80 (4.76)	3.72 (4.43)	4.42 (7.87)	13.55 (23.98)	0.06 (0.24)	0.89 (1.44)	0.22 (0.73)	0.24 (0.71)	0.58 (1.26)
Parents reported 0 min: will always feed immediately (%)	46.9	24.8	24.2	30.5	18.30	94.4	68.5	88.9	89.5	72.2

a Newborn period wording asked for parents’ plans after they got home, wording at later ages [in brackets] asked about current practices. Table 1 gives the numbers in each group at each age.

### Two-week (2W) assessments

Full written informed consent was obtained at a home visit when infants were 10–14 days old. Parents provided demographic information and the ISFAQ was repeated, re-worded to refer to current parenting practices. Researchers explained the Baby Day Diary (‘Diary’) and asked parents to keep this for 3×24 h days. The Diary is a validated, real-time parent-report measure of infant sleep, fuss/crying and awake-settled behaviour (Barr *et al*., 1988, 2005). Parents shade in successive behaviour periods against a scale of 5 min of time.

### Five-week (5W) assessments

ISFAQ current parenting and Diary measures were repeated. Following previous studies (Goodlin-Jones *et al*., 2001; Ball, 2007; Sitnick *et al*., 2008), researchers installed a self-focussing digital infrared video camera (Sony HDR-XR200VE) on a tripod directed at the infant’s night-time sleep location, allowing up to 13 h of continuous recording. Parents were instructed in camera use and asked to switch it on when they began settling their infant to sleep at night and off the following morning. They were asked to follow their usual night-time habits and could switch the equipment off at any time. The video was checked by researchers the following day and, if technical problems had arisen, one further attempt was made to obtain a recording.

### Three-month (3M) assessments

The ISFAQ, Diary and video measures were repeated and parents completed infant sleep questionnaires, which are analysed elsewhere (St James-Roberts *et al*., 2016). Because of extensive assistance with data collection, parents received high street shopping vouchers value £100 on returning the 3M data.

### Six-month (6M) assessments

After a telephone follow-up, the ISFAQ and sleep questionnaires were repeated and returned by mail. The study received Riverside Medical Research Ethics Committee approval (REC 09/H0706/11).

### Data coding and analysis

Data were coded in Excel spreadsheets by researchers using written manuals and trained to ⩾90% reliability. Parental report data were coded blinded. Video coders cannot remain blinded, but video and parental report data were coded by different researchers. The Excel data were exported to SPSS 22 (IBM, 2013) for analysis.

The start time, type and end time of each diary behaviour period was coded. Video coding rules were based on Anders’ methods and conventional definitions of infant behaviour states (Anders and Keener, 1985; Goodlin-Jones *et al*., 2001). Detailed descriptions are provided in St James-Roberts *et al*. (2015). In summary, the videos were coded to identify five behaviour period types: awake, sleep, indeterminate, out of view, and video turned off. Within each awake and sleep period, the times, frequency and durations of infant behaviours (sleep, drowsy, waking content, fuss/crying, feeding), of ‘direct parental contact’ (touching, holding or speaking to an infant) and of ‘checking’ (approach to an infant without direct contact) were coded. Maternal, paternal and other carer behaviours were coded separately, but are combined here for brevity. To confirm reliability, 20 videos and 20 diaries were duplicate-coded by independent coders. Video between-coder Pearson correlations ranged from 0.862 to 1.00. Diary overall coding agreement was 0.998.

## Results

### Descriptive figures for night-time parenting in London

#### Parental questionnaire (ISFAQ) measures

Most participants were white, highly educated and married or co-habiting (Table 1). The Bed-Sharing group contained proportionately fewer firstborn infants. Most General-Community infants (64%) but all Bed-Sharing infants were exclusively breast-milk fed at 3M. Most (93%) General-Community mothers planned their infants would usually sleep in cots in their bedroom, but 40–50% planned to, or occasionally did, bed-share with their baby for short periods (e.g., feeding). All but one Bed-Sharing infant still bed shared through the night at 3M.

As Table 2 shows, the intention to adopt evening routines to assist settling was equally common in both groups, usually involving bathing. To reduce analyses, it was excluded from further consideration.

The groups were compared at each age on response delay, feeding interval and settling method, using descriptive statistics and analysis of variance. As anticipated, approximately two-thirds of General-Community parents reported delaying responding to infant cries, or introduced an interval before feeding, at any one age (Table 2). Within the first three months (i.e., across 2W, 5W and 3M measures), mean response delays or feeding intervals ranged from 1.7 (SD 2.06) min to 4.4 (SD 7.9) min, and ⩾83% of parents reported responding, and feeding, within 5 min at each age. Settling methods were more mixed but, except at 10 days, most General-Community parents (59.4–85.2%) settled at a regular time (sometimes, usually, almost always or always).

In contrast, 79–95% of Bed-Sharing parents reported responding immediately to infant cries, and 68.5–94% fed immediately, at each age. Most (68–84%) Bed-Sharing parents planned to settle their infants when tired (usually, almost always or always).

These findings show that Bed-Sharing parents chose infant-cued parenting methods, whereas General-Community parents favoured limit-setting methods, at each age. Pearson’s two-tailed correlations showed that response delay and feeding interval in the General-Community infants were moderately correlated (e.g., 0.542 *P*<0.001 at 5W), whereas the settling method scores correlated weakly or non-significantly with response delay and feeding latency scores.

#### Video and diary validation of the parental questionnaire measures

On an average, 5W video recording started at 10:20pm and lasted for 9 h 12 min; 3M recording started at 9:44pm and lasted 10 h 11 min, providing ~1000 h of recording/age. St James-Roberts *et al*. (2015) provide descriptive details for the recordings. Little observation time was lost due to the video being switched off. However, General-Community parents removed infants from the video recording for more substantial periods than Bed-Sharing parents, largely for feeding and associated care (only nine of 99 General-Community infants were recorded feeding at five weeks). The respective ‘out of view’ means (SDs) were 100 min (85 min) versus 11 min (17 min) at 5W; 53 min (66 min) versus 5 min (21 min) at 3M. The amount of video ‘indeterminate’ time, when a baby’s behaviour could not be reliably scored, was greater in the Bed-Sharing than General-Community group, largely because a parent’s body or bed-clothing was obscuring the view. Respective means (SDs) were 63 min (100 min) versus 8 min (29 min) at 5 W; 20 min (47 min) versus 6 min (28 min) at 3M. These limitations of the video method are allowed for below.

## Response delay

Table 3 presents the findings. In the 5W videos, Bed-Sharing parents detected and responded to infant waking in a mean (SD) of 14 s (40.0 s), compared with a General-Community mean (SD) of 3 min 32 s (3 min 13 s). Bed-Sharing infants signalled for a mean (SD) of 2 s (8.0 s) before direct parental contact and 10 did not signal at all. General-Community infants signalled for 1 min 3 s on average (SD: 1 min 33 s.) before direct parental contact. All these 5W differences are highly statistically significant (Table 3) and confirm the parents’ questionnaire reports. The 3M differences were similar (Table 3).

**Table 3:** Comparison of the groups on video and diary measures of night-time parenting and infant behaviour at two weeks, five weeks and three months of age^a^

Groups	General-Community group			Bed-Sharing group		
Age	Two weeks	Five weeks	Three months	Two weeks	Five weeks	Three months
VIDEO: interval between infant waking and direct parental contact		0:03:32 (0:03:13)***	0:04:31 (0:05:35)***		0:00:14 (0:00:40)***	0:00:38 (0:01:16)***
VIDEO: interval between infant signalling and direct parental contact		0:01:03 (0:01:33)**	0:00:53 (0:01:17)**		0:00:02 (0:00:08)**	0:00:12 (0:00:39)**
VIDEO: % of waking periods including direct parental contact		26.70 (28.11)***	19.24 (20.49)***		89.03 (26.10)***	85.5 (27.17)***
VIDEO: % of sleeping periods including direct parental contact		06.2 (0.21)***	02.34 (11.33)***		76.16 (00.06)***	73.20 (31.83)***
VIDEO: infant behaviour state at the start of the video						
Infants asleep (%)		49.5^b^	39.6		50.0^b^	36.8
Infants drowsy (%)		11.1^b^	10.9		00.0^b^	0.00
Infants awake content (%)		24.2^b^	34.7		11.1^b^	36.8
Infants fuss/crying (%)		05.1^b^	9.9		11.1^b^	10.5
Infants feeding (%)		00.0^b^	01.0		22.2^b^	10.5
Infants indeterminate (%)		10.1^b^	04.0		05.6^b^	5.3
DIARY: number of feeds [mean (SD)]	5.23 (1.97)**	4.56 (1.77)***	3.30 (1.51)***	6.58 (2.20)**	6.25 (1.49)***	5.14 (2.07)***
DIARY: feed intervals [mean (SD)]	2:08:00 (0:42:00)**	2:29:00 (0:50:00)**	3:49:00 (2:00:00)**	1:37:00 (0:35:00)**	1:47:00 (0:25:00)**	2:15:00 (0:57:00)**

Figures are mean (SD) numbers or lengths of time in hours:minutes:seconds.

a VIDEO measures were collected only at five weeks and three months; DIARY measures at all three ages.Table 1 gives the numbers in each group for each method and age.

b Pearson *χ* ^2^ *P*<0.001.

All comparisons are between groups at the same age. ANOVA: *P<0.05; *P<0.01; ***P<0.001.

## Feeding interval

Because most General-Community infants were removed from the video-recording for feeding at 5W, diary data were used to measure feed intervals (the time between the end of one and start of the next feed). Table 3 gives the findings. Confirming the parents’ questionnaire reports, General-Community infants had longer feed intervals, and fewer feeds, than Bed-Sharing infants in the night at all three ages. There were no significant group differences in feed length.

## Settling methods

Settling at a scheduled time should reduce the number of infants already asleep or feeding when parents settled infants for the night. As Table 3 shows, around half the infants in the two groups were already asleep when video-recording began. However, General-Community infants were more likely to be drowsy or awake-content, and less likely to be feeding, when 5W, but not 3M, recording began. General-Community parents also spent much less time in direct contact with their babies throughout the night at 5W and 3M. These findings provide some support for the parents’ reported differences in settling methods, but these differences were less robust than those involving response delay and feeding interval.

### Amounts of infant distress associated with limit-setting parenting

**Figure 1:**
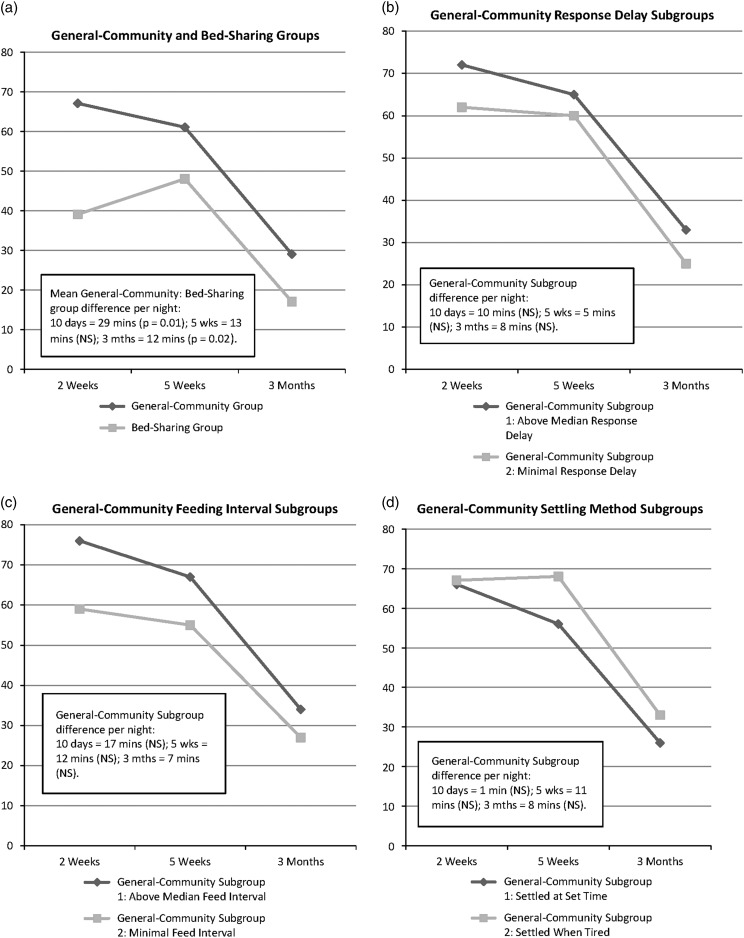
Diary-measured total minutes of infant distress (crying+fussing) per night (7pm–7am) at each age in the groups and General-Community subgroups: (a) General-Community and Bed-Sharing groups; (b) General-Community response delay subgroups; (c) General-Community feeding interval subgroups; (d) General-Community settling method subgroups.

The mean video-recorded time General-Community infants spent signalling without a direct parental intervention at 5W (1 min 3 s) was significantly longer than for Bed-Sharing infants (2 s). However, the video figures underestimate total distress because they overlook periods when infants were removed from video recording. Figure 1a shows the group Diary data. Bed-Sharing infants fuss/cried significantly less than General-Community infants, on average by ~29 min/night, at 2W (*F*=6.62, 1 df, *P*=0.01). At 5W this difference reduced to ~13 min/night and was not statistically significant. At 3M, total night-time fuss/crying in both groups approximately halved, giving a significant difference of ~12 min/night (*F*=4.95, 1 df, *P*=0.028).

As well as these group differences, the forgoing findings indicate variations within the General-Community group in limit-setting versus infant-cued parenting. To explore these, we used median splits to divide the overall General-Community group into two subgroups per age on each measure (response delay, feeding interval, settling method). This analysis compares General-Community infants whose parents adopted relatively limit-setting parenting (response delay >1 min at 2W and 5W, >1.5 min at 3M; feeding interval >2.5 min at 2W, >2 min at 5W and 3M; settling at a regular time sometimes-always at all three ages) with those who adopted more infant-cued methods on diary-measured distress at each age. Figures 1b–1d show the findings. None of the General-Community subgroup differences was statistically significant in these comparisons involving ~50 infants per subgroup. Because the response delay and feeding interval subgroups tended to fuss/cry more at each age (Figures 1b and 1c) the differences might be statistically significant in larger samples. However, the mean differences in distress are <1.5 min/h.

To ensure these findings were not distorted by the median-split method, we correlated the response delay, feeding interval and settling method scores with the diary measures of infant distress at each age in the General-Community group as a whole. None of these Pearson’s correlations was significant at 5W. Night-time fuss/crying correlated 0.244 (*P*=0.014) with feeding interval at 2W, and negatively with timed settling at 3M (−0.222; *P*=0.025). This negative correlation is counter-intuitive and the associations are weak, so that they may be chance findings. No other 2W or 3M correlations were significant. These findings confirm that concurrent relationships between parenting methods and infant distress are not strong within the General-Community group.

## Discussion

A recent review has questioned RCT evidence that ‘limit-setting’ parenting increases the number of infants with long night-time sleep periods at three to four months of age, arguing that this form of parenting risks increasing infant distress (Douglas and Hill, 2013). Following Medical Research Council (MRC, 2007) guidelines, the present study was designed to complement the RCT evidence, using video-recording and other methods to observe London infants’ night-time distress, and associated parenting behaviours, in their normal home environments. A General-Community group of 101 infants and parents, most of whom adopted limit-setting parenting methods, was compared with a group of 19 planned Bed-Sharing parents and babies, who adopted highly infant-cued parenting methods. This comparison group included parents who intended to bed-share from before their baby’s birth and did so consistently, unlike ‘reactive’ bed-sharers who respond to their infant’s night waking and signalling by switching to bed sharing (Germo *et al*., 2007). This group was smaller than intended, which needs to be taken account of when interpreting the findings. Assessments focussed on the first three months of age: the period when most western infants start to develop prolonged sleep periods at night.

The first finding was that London General-Community parents took longer to detect and respond to infant waking and signalling, and to begin feeding, compared with parents committed to infant-cued parenting. In most cases, the intervals reported were limited to 1–5 min and the average latency in General-Community parents’ responding to infant night-time waking observed in the videos was 3.5 min, during which infants fuss/cried for around 1 min. Such delays allow infants an opportunity to develop autonomous re-settling (Pinilla and Birch, 1993; St James-Roberts *et al*., 2001) and were much shorter than needed for ‘controlled crying’ treatments of infant sleep problems, so that these should not be equated. Bed-Sharing parents’ environmental settings may have facilitated their rapid responding. For instance, 10 of 19 Bed-Sharing infants received a direct parental contact before they fussed or cried at 5W, presumably because bed sharing allowed early detection of infant waking.

These differences in parental responsiveness were matched by differences in General-Community and Bed-Sharing infants’ total amounts of night-time (7pm–7am) distress. These differences, involving more distress in General-Community infants, were larger at two weeks (~30 min/night) than at five or 12 weeks of age (~12–13 min/night).

Our third finding was that, within the General-Community group, differences in infant night-time distress between subgroups where parents settled infants at a scheduled time rather than when tired, delayed responding rather than responding rapidly, or introduced an interval before feeding rather than feeding with minimal delay, were not large or statistically significant. For instance, the average reductions in infant distress where General-Community parents responded or fed with minimal delay (versus delaying response or including an interval before feeding) at night were <1.5 min of distress/h. In view of medical guidance that bed-sharing is unsafe, this finding may be particularly relevant to contemporary clinical practice. It is worth noting that these findings were based on subgroups of ~50 infants. Larger groups may reveal statistically significant differences but, even so, there is no reason to expect that the size of the differences has been underestimated here.

These findings may seem unremarkable since, on the face of it, neither London nor other western parents seem likely to leave their babies to cry for long periods. Even so, some reviewers have expressed concern about this issue (Douglas and Hill, 2013). The figures’ value is to provide specific descriptive evidence about limit-setting parenting as practiced in a western community which may counter some doubts about this form of parenting, provide reassurance, and help parents and professionals to make choices. Although some parents may consider that any infant distress should be avoided, others may judge that a night-time increase of around 1.5 min/h is justified by the potential benefits of this form of parenting.

The remaining question is what exactly those benefits are. Detailed analyses of the sleep-waking behaviours of the same cohort of infants assessed here found that stable limit-setting parenting, particularly inclusion of a short interval before feeding, led infants to have fewer and longer night-time sleep periods at 3M of age (St James-Roberts *et al*., 2016). This finding is consistent with the evidence from the majority of RCTs, but the longitudinal observation methods used can only indicate, not prove, causation. Moreover, the evidence from RCTs is not conclusive at the moment.

Because of these uncertainties, we do not think that limit-setting parenting should be recommended prescriptively by contemporary health services. Instead, the present findings and controversies can be communicated to parents to help them to make informed choices that take account of their preferences and circumstances. The findings highlight the need for further research to clarify how relatively subtle differences in parenting promote infant sleep-waking development – and the metabolic adaptations involved in it – during this key period for sleep-waking development. The existing findings do not support the long-standing assumption that breast-milk constituents require three-month-old infants to wake frequently at night (St James-Roberts *et al*., 2015).

Longer-term research is also needed. The RCT findings imply that limit-setting parenting should result in less infant night-time distress and improved sleeping after three months of age, while improved sleeping should help to support healthy infant development (El-Sheikh and Sadeh, 2015). However, there is little direct evidence to confirm these benefits or to establish the long-term benefits of infant-cued parenting. Rather than debating the ‘best’ form of parenting, overall cost-benefit analyses should be especially helpful for parents and practitioners wishing to make evidence-informed choices.
